# Flame-Retardant Properties of a Styrene-Vinyl Tetrazole Copolymer Additive in an LDPE/EVA Blend

**DOI:** 10.3390/polym17212933

**Published:** 2025-10-31

**Authors:** Karla Fabiola Rodríguez Ramírez, Jesús Francisco Lara Sánchez, Orlando Castro Reyna, Pedro Espinoza Martínez, Jesús Alejandro Espinosa Muñoz, José David Zuluaga Parra, Rachel Faverzani Magnago, Saul Sanchez Valdés, Luciano da Silva

**Affiliations:** 1 Department of Transformation Processes, Applied Chemistry Research Center, Blvd. Enrique Reyna H. 140, Saltillo 25294, Mexico; karla.rodriguez.d22@ciqa.edu.mx (K.F.R.R.); jesus.lara@ciqa.edu.mx (J.F.L.S.); orlandocastroreyna@gmail.com (O.C.R.); pedro.espinoza.d22@ciqa.edu.mx (P.E.M.); alejandro.espinosa@ciqa.edu.mx (J.A.E.M.); saul.sanchez@ciqa.edu.mx (S.S.V.); 2 Rothamsted Research, West Common, Harpenden AL5 2JQ, UK; josedavidzuluaga@gmail.com; 3 Sustainable Technologies Research Group, Federal University of Santa Catarina, Florianópolis 88040-900, SC, Brazil; rachelfaverzanimagnago@gmail.com

**Keywords:** flame retardants, tetrazole, LDPE/EVA, intumescence, ammonium polyphosphate

## Abstract

In this work, we report the effect of combining styrene-vinyl tetrazole copolymer (StVTz) and ammonium polyphosphate (APP) on the thermal degradation, mechanical properties, flame retardancy, and char formation of low-density polyethylene with ethyl vinyl acetate (LDPE/EVA) composites. The tetrazole heterocycle exhibits high thermal stability (>200 °C), and during its thermal decomposition, it releases non-toxic nitrogen gas. Its degradation generates reactive species capable of cross-linking the polymer chains, thereby promoting the formation of a protective char layer. To evaluate the influence of composition on the intumescent flame-retardant (IFR) properties of LDPE/EVA blends, different concentrations of APP and StVTz additives were incorporated. The composites were prepared in an internal mixer (Brabender Intelli-Torque Plasti-Corder). Test specimens were obtained by compression molding and subsequently cut into appropriate shapes for each analysis. Thermal stability was studied by thermogravimetric analysis (TGA) and differential scanning calorimetry (DSC). Mechanical properties were evaluated by tensile testing. Morphology of cone calorimetry (CC) residues was examined using SEM. Flammability properties, studied using CC, revealed a 70% reduction in the peak heat release rate (pHRR) and a 48% reduction in the total heat release (THR) compared to the neat LDPE/EVA blend. These results indicate that StVTz and APP act synergistically to improve the flame-retardant properties of LDPE/EVA.

## 1. Introduction

Polyethylene is one of the most widely used plastics in the world, with applications in diverse sectors; however, PE is highly flammable [1]. Flame retardants (FRs) are designed to slow down or suppress flames on a material. They are incorporated when products fail regulatory fire safety tests or must comply with fire performance standards. The plastics sector is the largest consumer of FRs [2]. Low-density polyethylene (LDPE) is one of the most produced polymers worldwide, with key applications in the cable industry [3]. FRs are essential to meet requirements such as low smoke generation, reduced toxic gas release, and limited flame spread in accordance with modern regulations [4]. FRs can be incorporated through physical or chemical treatments [5]. The addition of FR additives during polymer melt processing is considered a simple and cost-effective strategy. Phosphorus-based FRs are the most common in PE systems [6], including red phosphorus, phosphines, phosphates, and ammonium phosphate. Nitrogen-based FRs have also been widely used, such as melamine (MLM, 67 wt.% N) which has excellent thermal resistance [7]. Melamine is extensively used in industrial plastics, coatings, wood-based panels, paints, and surface finishes. Although minimal amounts of melamine may migrate into food during normal use, such exposure is generally considered low risk. However, exposure to high concentrations of melamine has been shown to pose toxicological risks (UN, News, 2010) [8], leading to its official classification in the European Union as a suspected carcinogen and organ-damaging substance upon prolonged exposure [9]. Its persistence in the environment also raises concerns as a potential groundwater contaminant [10].

Phosphorus-based intumescent flame retardants are effective due to their ability to form a protective carbon layer in the condensed phase mechanism [11]. Among them, organophosphorus polymers are the most suitable systems to prevent fire propagation [12]. For instance, Xie et al. reported that metal chelates in LDPE systems enhanced the efficiency of IFR by acting as radical inhibitors and promoters of carbonization. Furthermore, cross-linking increased the stability of APP and made more phosphorus available for phosphorylation and char formation, thereby improving the protecting effect of the resulting char [13]. Liao et al. studied a system of LDPE with piperazine-modified ammonium polyphosphates, achieving efficiency in flame retardancy, a V-O rating, and a limiting oxygen index (LOI) of 33% [14]. Wang et al. developed a phosphorus-tetrazole compound incorporated into an epoxy resin, where tetrazole decomposition released nitrogen gas, inducing intumescence and diluting flammable gases [15]. Tetrazoles are aromatic heterocycles composed of one carbon atom and four nitrogen atoms (one pyrrole-like and three pyridine-like) [16]. They are widely studied scaffolds with applications in various fields, including functional materials for solar cells [17,18,19], the materials industry [20], and military applications [21]. Their thermal decomposition produces nitrogen gas (N_2_) and a reactive intermediate known as nitrilimine. This species can participate in 1,3 dipolar cycloaddition reactions with alkenes, imines, alkynes, and nitriles [22], thus enhancing char formation on the polymer surface.

The combination of tetrazole derivatives with APP is therefore of great interest due to their potential synergistic action. However, reports on tetrazole-based compounds in FRs systems remain scarce. In this study, we present the synthesis of a styrene–vinyl tetrazole copolymer, its incorporation into LDPE/EVA in combination with APP, and the evaluation of their structural, thermal, mechanical, and flame-retardant properties.

## 2. Experimental Section

### 2.1. Materials

Hydrochloric acid (J.T Baker, Phillipsburg, NJ, USA), methylene chloride (99%, Aldrich, Burlington, MA, USA), and methanol (99.8%, Aldrich) were used without prior purification. Styrene (St, 99%, Aldrich) was purified with inhibitor removers for butylcatechol. Acrylonitrile (AN, >99%, Aldrich) was purified with inhibitor removers for hydroquinone and stored under refrigeration. Benzoyl peroxide (BPO), St, and AN were stored in the dark at approximately 4 °C before use. BPO, sodium azide (NaN_3_, >97%), and ammonium chloride (NH_4_Cl, >99.5%) were purchased from Sigma-Aldrich (St. Louis, MO, USA) and used without purification. APP was provided by Hangzhou JLS Flame Retardants Chemical Co. (Hangzhou, China), lot N. 2019011602, and used as received. Low-density polyethylene (LDPE) PE-20020X was purchased from PEMEX (Mexico City, Mexico) with a melt flow index of 2.2 g/10 min and used without prior purification. Ethylene Vinyl Acetate copolymer (ELVAX 360) was purchased from Dow Chemical (Midland, MI, USA) and used as received.

### 2.2. Synthesis

#### 2.2.1. Synthesis of Poly(styrene-co-acrylonitrile) (EstAcn)

EstAcn copolymer was prepared by bulk free radical polymerization. Acrylonitrile (101.64 g, 1.92 mol) was placed in a 1 L three-necked flask (Aldrich, Burlington, MA, USA) equipped with mechanical stirring, followed by BPO (0.48 g, 0.002 mol). The mixture was stirred for 30 min at 70 °C, after which Styrene (99.16 g, 0.95 mol) was added. The reaction was maintained at 85 °C for 3 h. After completion, the mixture was cooled to room temperature, dissolved in CH_2_Cl_2,_ and poured into 100 mL of methanol. The white solid obtained was filtered, washed three times with cold methanol, and dried under vacuum. Yield 87%.

#### 2.2.2. Synthesis of Copolymer Styrene-Vinyl Tetrazole (StVTz)

In a 1 L three-necked flask equipped with mechanical stirring and heating, EstAcn (100 g, 1.43 mol) and N,N-dimethylformamide (DMF, 0.4 L) were added. The mixture was heated to 120 °C until complete dissolution. Sodium azide (71.5 g, 1.1 mol) and ammonium chloride (58.85 g, 1.1 mol) were divided into three equal portions (23.83 g of sodium azide and 19.61 g of ammonium chloride each). The portions were added sequentially at two-hour intervals. The mixture was stirred and heated for 72 h and then cooled to room temperature and poured into 500 mL of ice-cold water. The mixture was acidified with HCl solution to pH 2, and the resulting precipitate formed was filtered, washed with cold water, and dried in a vacuum oven at 60 °C for 8 h. The obtained product was recrystallized from ethanol to afford a white solid. The yield was 95%.

### 2.3. Preparation of LDPE/EVA Composites

LDPE/EVA composites containing APP and StVTz were prepared in an internal mixer (Brabender, Duisburg, Germany, Intelli-torque Plasticorder) at 180 °C and 60 rpm. First, the LDPE/EVA pellets were added and melted for 5 min. Subsequently, APP was added and mixed for 5 min, followed by the addition of StVTz for another 5 min. The resulting mixture was cooled, ground, and characterized. To investigate the individual contribution of StVTz and APP, five different formulations were prepared, as listed in Table 1. Test specimens were obtained by compression molding at 180 °C and 4 tons for 5 min. These samples were used for mechanical and thermal analyses. The composites are designated as LDPE/EVA-X-Y, where X denotes the APP content and Y the StVTz content (Table 1).

### 2.4. Characterization

To evaluate the structural, thermal, mechanical, and flame-retardant properties of the developed composites, a series of standardized characterization techniques were employed.

Fourier Transform Infrared Spectroscopy (FT-IR): FT-IR spectra were obtained on a Bruker VERTEX 70v spectrometer, with 128 scans at 4 cm^−1^ resolutions, over the range of 4000 to 500 cm^−1^.

Nuclear Magnetic Resonance (NMR): ^1^H NMR spectra were recorded on a Bruker Avance III, 400 MHz spectrometer, and ^13^C NMR spectra were recorded at 125 MHz using the same equipment.

Thermogravimetric Analysis (TGA): Thermograms were performed on a Linseis STA PT 1000 in the range of 50–800 °C at a heating rate of 10 °C/min under air atmosphere.

Differential Scanning Calorimetry (DSC): This was performed using a TA Instruments Q2000 calorimeter in the range of 0–200 °C at 10 °C/min under nitrogen atmosphere.

Mechanical Properties (Tensile Strength): Tensile tests were determined using an MTS Criterion Model 43 universal testing machine (5 kN) at 50 mm/min. Type V specimens (2 mm thickness) were prepared according to ASTM D638-Standard Test Method for Tensile Properties of Plastics [23]. 

Scanning electron microscopy (SEM): Morphological analysis of CC residues was performed using a JEOL SM-6000 microscope at 15 kV under high vacuum, with magnifications of 200× and 700×.

Cone Calorimeter (CC): Flame retardant performance was evaluated using a cone calorimeter (ASTM E1354). Samples of 100 mm × 100 mm × 2 mm were exposed to a heat flux of 35 kW/m^2^ at 25 mm distance, with a nominal duct flow of 24 L/s and a correction factor of 0.043411.

UL-94 Horizontal Burning Test (UL-94 HB): This test was conducted in accordance with the ASTM D635 standard.

Limiting Oxygen Index (LOI): This was determined following ASTM D2863—Standard Test Method for Measuring the Minimum Oxygen Concentration to Support Candle-Like Combustion of Plastics (Oxygen Index) [24], to establish the minimum oxygen concentration required to sustain combustion.

## 3. Results and Discussion

### 3.1. Chemical Structure of Copolymers

**Figure 1:**
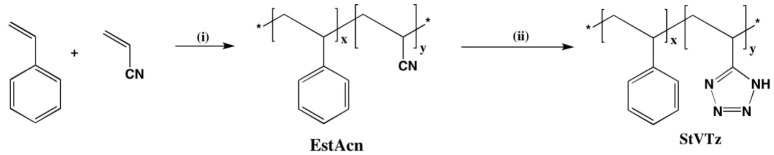
Structure of copolymer styrene-vinyl tetrazole (StVTz). (i) = BPO, 85 °C, 3 h; (ii) = NH_4_Cl, NaN_3_, 120 °C, DMF, 72 h. (Asterisks represent continuation of a polymer chain).

StVTz was synthesized by adapting the methodology reported in the literature (Figure 1) [25].

The styrene–acrylonitrile copolymer (EstAcn) was synthesized under radical polymerization conditions at a ratio of 55% styrene and 45% acrylonitrile (molar ratio). The resulting copolymer was characterized by FTIR, ^1^H NMR, and ^13^C NMR.

The FT-IR spectrum of EstAcn copolymer displays the characteristic C_sp2_-H stretching band of styrene between 3100 and 3000 cm^−1^, along with a C=C stretching band at 1607 cm^−1^. Bands at 2950–2850 cm^−1^ are attributed to the asymmetric and symmetric stretching vibrations of C_sp3_-H bonds (from CH_2_ and CH groups) in the aliphatic backbone. Additionally, a sharp absorption band at 2243 cm^−1^ is observed, which is attributed to the nitrile (-C≡N) functional group (Figure S1, Supplementary Data). The EstAcn copolymers were subsequently converted into the StVTz copolymer. The FT-IR spectrum of the StVTz copolymer (Figure S1, Supplementary Data) confirms the formation of the tetrazole ring. A broad absorption between 2500 and 3500 cm^−1^ corresponds to tetrazole stretching vibrations. The signal at 1659 cm^−1^ corresponds to C=N stretching of the tetrazole system [26]. The disappearance of the characteristic band of the nitrile group (at 2237 cm^−1^) and the broadening of the signals in the region between 2600 and 3500 cm^−1^ confirm complete tetrazole formation.

The ^1^H and ^13^C NMR spectra of EstAcn are shown in Figure S2 (Supplementary Data). In the ^1^H-NMR spectrum, a broad signal appears between 1 and 3 ppm, corresponding to methylene (CH_2_) and methine (CH) protons in the copolymer backbone. From 6.5 to 7.5 ppm, the protons of the aromatic system are observed, corresponding to the part of the styrene group in the copolymer. In the ^13^C-NMR spectrum, quaternary carbons of the benzene ring are observed between 138 and 142 ppm. The other types of sp^2^ hybridization carbons of the aromatic system are observed as multiple signals between 127 and 130 ppm. The nitrile carbon is observed between 119 and 121 ppm.

The ^1^H NMR spectrum of StVTz (Figure S2, Supplementary Data) shows multiplet signals between 1 and 3 ppm from C_sp3_-H protons in the aliphatic chain. The aromatic system appears as a multiplet between 6.5 and 7.5 ppm. In the ^13^C NMR spectrum, aromatic carbons of styrene units appear at 126, 128, and 129 ppm, while a new small peak at 161 ppm is assigned to the quaternary carbon of the tetrazole group.

### 3.2. Thermal Characterization of Copolymers

Figure S3 (Supplementary Data) shows the thermal degradation curve of EstAcn and StVTz. It can be observed that EstAcn presents a single weight loss of 93.36% of the initial mass. This degradation process begins at 323 °C. The literature reports the decomposition of these copolymers between 265 and 440 °C [26]. In contrast, the TGA curve of the StVTz copolymer reveals three distinct weight losses. The first weight loss, occurring between 90 °C and 170 °C (7%), is attributed to the evaporation of residual solvents. The second, between 208 and 290 °C (16.87%), corresponds to tetrazole decomposition with N_2_ release. The final weight loss, corresponding to 59.84% of the total mass, begins at 307 °C and is attributed to the degradation of the polymer backbone.

The EstAcn copolymer has a Tg of 116 °C. Acrylonitrile content increases chain rigidity, raising Tg. StVTz shows a Tg of 165 °C (Figure S4, Supplementary Data), higher than EstAcn, due to increased rigidity and stronger intra- and intermolecular interactions from tetrazole groups [27].

### 3.3. LDPE/EVA Composites

#### 3.3.1. Chemical Characterization

**Figure 2:**
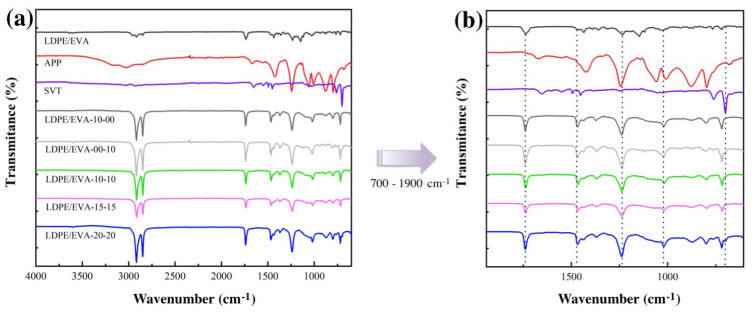
FT−IR spectra of LDPE/EVA composites containing StVTz and APP. (**a**) FT−IR spectra of region between 700 and 4000 cm^−1^; (**b**) Broadening of the spectrum in the region between 700 and 1900 cm^−1^.

The LDPE/EVA compounds containing StVTz and APP were chemically characterized by FT−IR spectroscopy (Figure 2a). The stretching vibration signals of the Csp^3^-H bonds from the ethylene segments were observed at 2914 cm^−1^ and 2842 cm^−1^, attributed to the asymmetric and symmetric vibration modes, respectively. Additionally, the methylene bending vibration was detected at 1470 cm^−1^. The characteristic vinyl acetate signals are identified by the stretching vibration of the carbonyl group (C=O) at 1734 cm^−1^ and the symmetric stretching of the C-O-C group at 1240 cm^−1^ and 1023 cm^−1^ [28,29]. The APP spectrum exhibited characteristic peaks in the 3300–3000 cm^−1^ region and at 1420 cm^−1^, attributed to the N-H stretching of the ammonium group. The stretching vibration of the P=O bond appeared at 1240 cm^−1^, while its symmetric stretching was observed at 1055 cm^−1^, and the phosphate group’s (${PO}_{4}^{-3}$) vibration at 1016 cm^−1^ (Figure 2b) [30]. In the case of StVTz, we observe the stretching signal of the Csp^2^-H from the aromatic system at 3010 cm^−1^. The stretching vibration of Csp^3^-H appears at 2912 cm^−1^. A broad band at 1655 cm^−1^ is attributed to C≡N stretching. N=N and C=C vibrations are observed at 1602 and 1547 cm^−1^, respectively. These signals are also present in the compounds containing StVTz, although with lower intensity due to reduced concentration and band overlap among the different components.

In composites, the symmetric and asymmetric stretching vibrations of P-O were broadened at 1050 cm^−1^. The change in intensity of this band may be due to interactions between APP and the LDPE/EVA matrix. Due to the crystallinity of polyethylene, the characteristic bands at 1464 and 719 cm^−1^ exhibit splitting, and additional peaks are seen at 1473 and 760 cm^−1^. Polyethylene crystallinity can be determined from the ratio of the 760 to 720 cm^−1^ peaks (ASTM D5576 - Standard Practice for Determination of Structural Features in Polyolefins and Polyolefin Copolymers by Infrared Spectrophotometry (Last Updated: 10 February 2021-https://www.astm.org/) [28]. Changes in this ratio suggest a variation in the crystallinity induced by the incorporation of additives.

#### 3.3.2. Thermal Characterization

**Figure 3:**
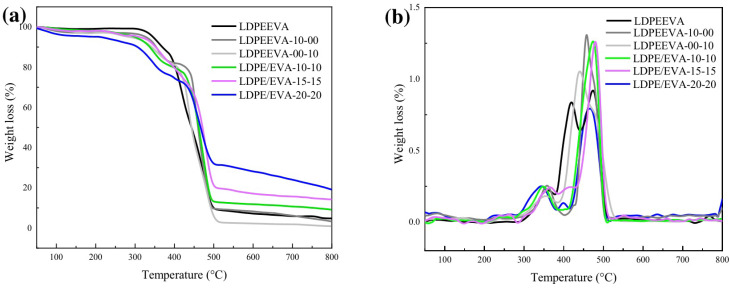
TGA (**a**) and DTG (**b**) curves under air of LDPE/EVA and its composites.

TGA curves are shown in Figure 3a and DTG curves are shown in Figure 3b. The LDPE/EVA blend shows an initial decomposition temperature of 300 °C, corresponding to the degradation of vinyl acetate into acetic acid [31]. LDPE backbone decomposition starts at 385 °C [32,33], and the maximum rate of weight loss (T_max_) is at 474 °C. All composites display a similar T_max_ value (Table 2).

The incorporation of FR additives can influence the thermal behavior of the polymer matrix. IFR must decompose earlier than the polymer to produce reactive species that form a protective char layer. Additives StVTz and APP decompose at temperatures between 210 °C and 280 °C. Thus, TGA effectively reveals their influence [34].

For LDPE/EVA-10-00 formulation, we observed a slight weight loss over the temperature range of 278 °C associated with the degradation of APP, which releases water and ammonia, along with the formation of crosslinked polyphosphoric acids. Above 500 °C, APP further decomposes into phosphoric acid, metaphosphoric acid, and polymetaphosphoric acid [35,36]. The release of phosphoric acid and volatile gases such as NH_3_ and N_2_ contributes to the swelling of the char structure [33].

LDPE/EVA composites containing both APP and StVTz exhibit three distinct weight losses. The first stage occurs from 200 °C to 250 °C and is due to the decomposition of the tetrazole group. A second weight loss is observed from 280 °C due to the degradation of APP. As the StVTz content increases, a greater weight loss is observed in the lower temperature range (from 200 °C), indicating its contribution to early-stage degradation.

The residues at 650 °C for LDPE/EVA, LDPE/EVA-10-00, and LDPE/EVA-00-10 were 6%, 7%, and 2%, respectively, implying low charring ability. In contrast, composites containing both APP and StVTz showed significantly higher char residues (>10%), suggesting their synergetic role in char formation. The formulation LDPE/EVA-20-20 exhibited the highest char residue (26% at 650 °C), implying that the system combines a carbon source, an acid source, and a blowing agent, leading to enhanced char formation through phosphorus–nitrogen synergy.

**Figure 4:**
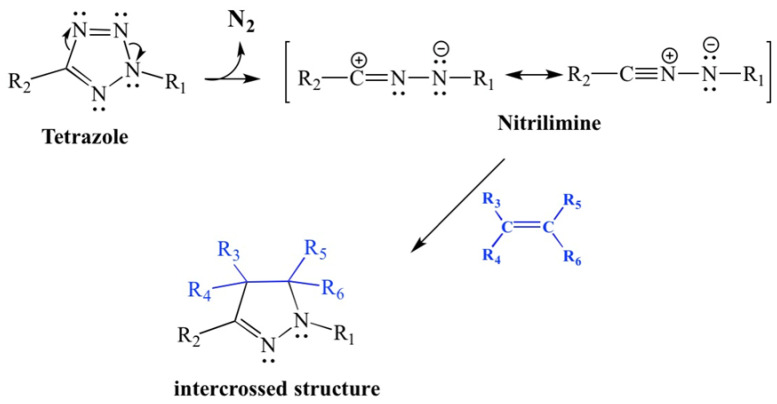
Thermal fragmentation of 2,5-disubstituted tetrazoles [29].

Tetrazole is proposed as an intumescent agent through thermal fragmentation, releasing N_2_ and forming a reactive nitrilimine intermediate, which leads to the 1,3 cycloaddition with alkenes, imines, nitriles, and alkynes (Figure 4) [37].

The nitrilimine intermediate will promote char formation and, with nitrogen liberation, encourage the system’s expansion along with the APP.

**Figure 5:**
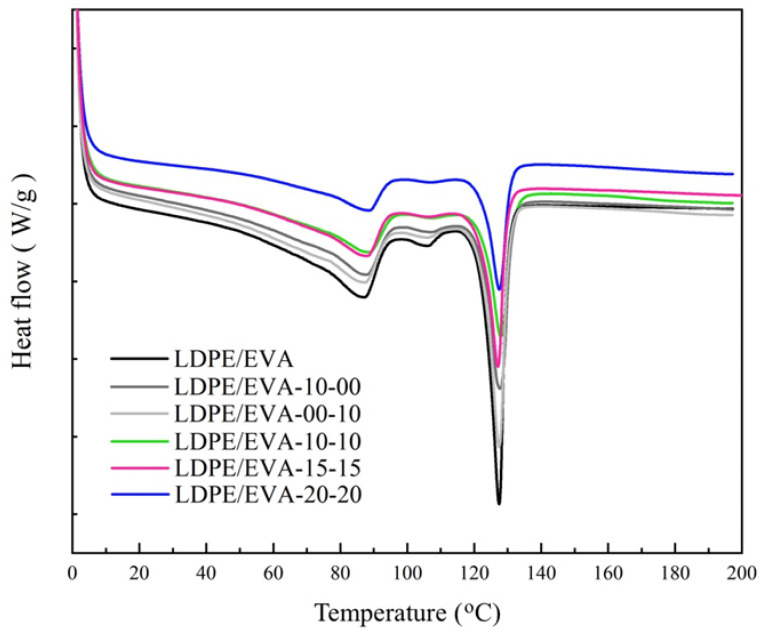
DSC curves of LDPE/EVA and LDPE/EVA composites.

DSC thermograms of LDPE/EVA composites are shown in Figure 5 and were used to evaluate the effect of FR additives on the melting point and crystallinity of LDPE/EVA. The crystallization temperature of the LDPE/EVA composites showed no remarkable changes compared to neat LDPE/EVA, and the melting point during the second heating remained constant at 127 °C in all formulations. Overall, the incorporation of FR at loadings of 10%, 15%, and 20% did not significantly change the thermal properties of LDPE/EVA.

However, crystallinity (X_c_) decreased with additive content (Table 3). While the neat LDPE/EVA exhibited a crystallinity of 7.9%, the value dropped to 3.7% in LDPE/EVA-20-20 formulation. Although this reduction did not affect the FR performance, it may have an impact on the mechanical properties [28,31,32,33].

The fillers do not function as effective nucleation sites, resulting in minimal change to the crystallization temperature (Tc). However, they hinder polymer chain mobility and limit the portion of the polymer capable of forming ordered crystalline regions, causing a pronounced reduction in crystallinity as the additive content increases [38,39].

#### 3.3.3. Mechanical Properties

LDPE/EVA exhibits higher elongation at break values compared to the composites (Table 4). The addition of FR additives results in a decrease in tensile strength as the additive content increases, while the modulus increases with the incorporation of additives. The presence of APP and StVTz leads to an increase in the stiffness of the LDPE/EVA matrix, with the most significant changes observed in the elongation at break, especially at higher loadings, with an increase in the Young’s Modulus (50%, 90%, and 170%). In contrast, the changes in tensile strength were less pronounced (52%, 50%, and 52%, respectively) compared to the neat LDPE/EVA. According to the results of LDPE/EVA-10-00 and LDPE/EVA-00-10, it is evident that StVTz has a greater influence on the reduction in tensile strength and elongation at break. This trend can be attributed to the fact that StVTz interrupts the crystallinity of LDPE/EVA in major grades compared to APP [40].

#### 3.3.4. Flame Retardant Performance

**Figure 6:**
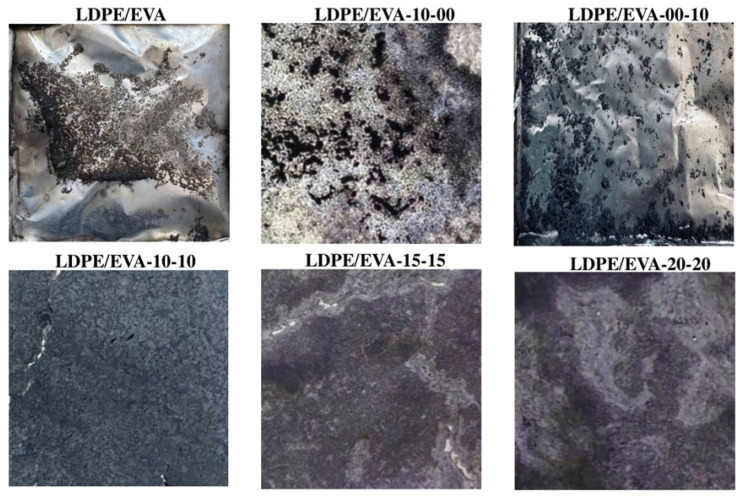
Images of the combustion results of LDPE/EVA and composites.

**Figure 7:**
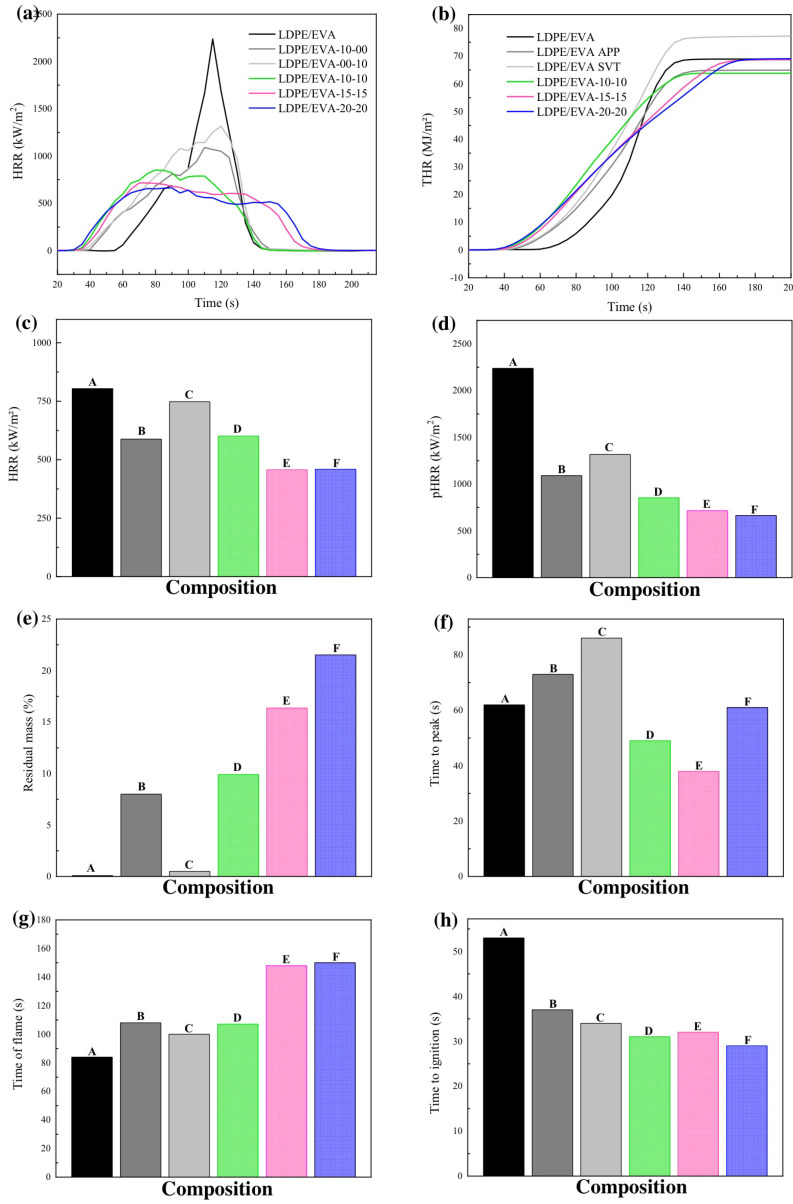
Flame retardant performance parameters of LDPE/EVA and its composites. (**a**) Heat release rate (HRR) curve; (**b**) total heat release (THR) curve; (**c**) HRR values; (**d**) pHRR values; (**e**) residual mass values; (**f**) time to peak heat release (TpHRR); (**g**) time of flame (TOF); (**h**) time to ignition. In (**c**–**h**), A represent the composition LDPE/EVA, B represents the composition LDPE/EVA-10-00, C represents the composition LDPE/EVA-00-10, D represents the composition LDPE/EVA-10-10, E represents the composition LDPE/EVA-15-15, and F represents the composition LDPE/EVA-20-20, respectively. Data obtained from CC tests.

To evaluate the flame retardant performance of LDPE/EVA and its composites, CC and UL-94 HB tests were conducted. The neat LDPE/EVA burns uniformly with an intensive flame that consumes the entire sample and leaves no residue after combustion, as shown in Figure 6. The heat release rate (HRR) and total heat release (THR) curves are presented in Figure 7, with the corresponding values listed in Table 5.

A notable reduction in HRR was observed with increasing amounts of APP and StVTz additives. For instance, the HRR of the LDPE/EVA-20-20 composite decreased by 43% compared to the neat LDPE/EVA. Similarly, a significant reduction in peak heat rate release (pHRR) was observed, with LDPE/EVA-20-20 showing a 70% decrease. The time to peak heat release rate (TpHRR) also exhibited a decreasing trend compared to the LDPE/EVA matrix.

In contrast, THR did not vary as significantly as the HRR parameters. Slight reductions were noted for LDPE/EVA-10-00 and LDPE/EVA-10-10, with decreases of 4% and 7%, respectively. The time to ignition (TTI) also showed a decreasing trend, likely due to the presence of phosphate groups in the additives, which promote crosslinking reactions that tend to anticipate thermo-degradation [41]. Also, the additives may increase the viscosity of the polymer melt, reducing dripping and leading to a more rapid rise in surface temperature, shortening the ignition time [42]. This trend is also supported by TGA data, where an increase in additive percentage leads to a lower initial decomposition temperature (T_d5_). The earlier decomposition of IFR allows it to react with the polymer matrix, promoting the formation of a protective char layer. Char residue increased with additive loading, and it was the highest in LDPE/EVA-20-20.

In the UL 94 horizontal burning test (Table 5) all samples achieved HB classification. Dripping was reduced in LDPE/EVA-10-10, LDPE/EVA-15-15, and LDPE/EVA-20-20 compared to neat LDPE/EVA.

The limiting oxygen index values (Table 5) increased with a higher additive content. The LDPE/EVA-20-20 achieved an LOI of 24%, which is considered self-extinguishing, in contrast to the neat polymer LDPE/EVA, which had an LOI of 17%, corresponding to high flammability.

**Figure 8:**
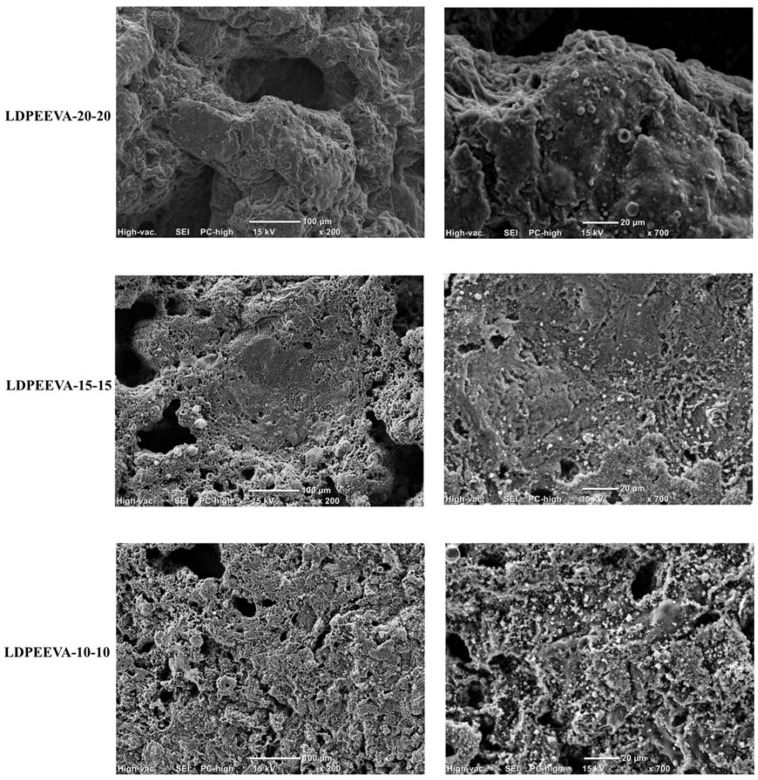
SEM images of cone calorimetry residues of LDPE/EVA-10-10, LDPE/EVA-15-15, and LDPE/EVA-20-20. Magnification: 200× and 700×.

The SEM images of the CC residues are shown in Figure 8. The char formed in the LDPE/EVA-10-10 appeared thin, no swelling was observed, and it presented a porous structure with agglomerates. The large gaps and holes observed allowed oxygen to penetrate deeper into the material and resulted in lower flame retardancy compared to LDPE/EVA-15-15 and LDPE/EVA-20-20 [27]. This may be attributed to poorer dispersion of additives.

At a higher additive percentage, the chars became denser and more compact. LDPE/EVA-20-20 exhibited the densest char with minimal agglomerates, as well as reduced porosity, consistent with improved pHRR reduction.

EDS analysis results are presented in Table 6. Residues are mainly composed of C, O, N, and P. These elements contribute to the formation of a cross-linked network within the char. The decomposition of APP and StVTz further promotes the formation of this protective char.

In all three composites, the presence of phosphorus and nitrogen was detected, confirming the contribution of APP and StVTz to char formation. In the LDPE/EVA-20-20 composite, a higher oxygen content suggests more compact and effective char.

The char layer acts as a physical barrier between the polymer and the flame, consequently protecting the underlying materials. In the LDPE/EVA-15-15 composite, a higher carbon content may suggest the formation of P–O–C and P–N–C structures that can improve the stability of the char. These cross-linked structures facilitate the formation of char through aromatization at high temperatures [34]. Additionally, the release of NH_3_ and N_2_ from StVTz can dilute combustible gases and participate in the FR mechanism.

The addition of intumescent-type flame retardants and synergistic fillers in LDPE/EVA systems has been shown to substantially reduce pHRR, with reported reductions between 40 and 50% depending on system and loading. For instance, Liu et al. reported that modified EVA systems exhibited reductions in THR and PHRR of 16.1% and 47.5%, respectively, compared to pure EVA [43]. Similarly, Monásček et al. observed improved flame retardancy after the addition of boric acid to APP formulations, with an increase in LOI up to 32%, although without any improvement in THR [44]. In our work, the best formulation reduced pHRR from 2237 kW/m^2^ for pure LDPE/EVA to 664 kW/m^2^ in an LDPE/EVA system with APP and a tetrazole-derivative copolymer, corresponding to a 70% reduction relative to the neat blend. The LOI likewise increased from 17% (LDPE/EVA) to 23–24% for the optimized formulations. Moreover, the incorporation of 20% additives into LDPE/EVA matrix resulted in an 8% decrease in THR. These results indicate a meaningful improvement in flame retardant performance, comparable with reports in the literature. However, despite the reductions in heat release, the UL-94 classification remains HB, indicating that while the additives effectively reduce heat release and promote char formation, further formulation or synergists are required to achieve V-0 ratings reported in some studies.

#### 3.3.5. Flame Retardant Mechanism of LDPE/EVA Composites

To study the mechanism, Thermogravimetric Infrared (TG-IR) analysis was performed. This allowed simultaneous monitoring of mass loss and identification of volatile species products during decomposition.

For phosphorus–nitrogen-based FRs, in the literature, we find two approaches that can act as flame retardants, namely, the gas phase and the condensed phase [41]. In the gas phase, the polymer matrix will produce radicals such as H· and CH3· at high temperatures, while APP generates PO· and PO_2_·. These radicals will combine with each other and terminate the active radicals to achieve the purpose of interrupting and inhibiting the combustion of polymer, thereby improving flame retardancy [45]. Meanwhile, in the condensed phase, acids, e.g., phosphoric acid (H_3_PO_4_), metaphosphoric acid (HPO_3_), and polymetaphosphoric acid decomposed from APP, will contribute to the dehydration of polymer into carbon [46]. This carbon layer can block the generation of new radicals by limiting the diffusion of oxygen in the combustion environment and can protect the underlying polymer from further combustion. At the same time, thermal decomposition reactions will lead to the release of nitrogen, ammonia, and water that dilute the combustion gases and decrease the matrix temperature, thus producing a flame-retardant effect [47,48].

**Figure 9:**
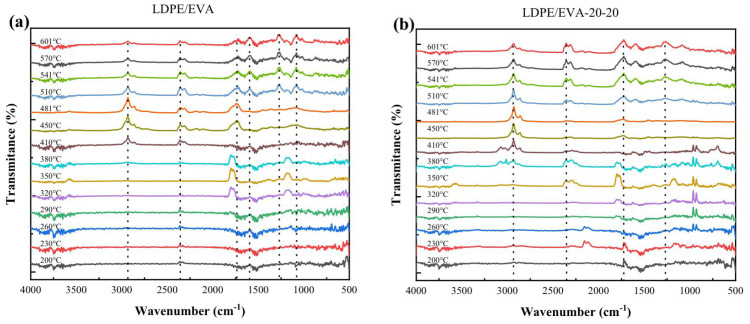
The TGA–IR spectra at different temperatures of (**a**) LDPE/EVA and (**b**) LDPE/EVA-20-20.

For neat LDPE/EVA (Figure 9a), degradation proceeds in two main stages. The first, starting near 300 °C, corresponds to the elimination of acetic acid from vinyl acetate units. FT-IR spectra reveal characteristic absorptions at 1800 cm^−1^ (C=O stretching), 1184 cm^−1^ (C–O stretching), and 3593 cm^−1^ (O–H stretching), confirming acetic acid release. The second stage, above 380 °C, involves polyethylene backbone scission with the liberation of volatile hydrocarbons (Csp^3^–H stretching at 2935 cm^−1^) and CO_2_ (2362 cm^−1^). Upon further heating (440 °C), a band emerges at 1748 cm^−1^, corresponding to carbonyl (C=O) groups from carboxylic acids, produced in the late-stage degradation of the residual polymer chain.

For LDPE/EVA-20-20 (Figure 9b), three weight losses are seen. These processes started at 200 °C, 300 °C, and 380 °C. The initial stage is associated with tetrazole decomposition, accompanied by N_2_ (undetectable by FT-IR). The second stage corresponds to APP degradation, water, ammonia (NH_3_ at 1509 cm^−1^), and condensed-phase acids (P=O and P–O–P at 965 cm^−1^), which participate in char formation. We can observe CO_2_ liberation (2359 cm^−1^) during these processes. At 380 °C, LDPE/EVA degradation begins, releasing acetic acid (1734 cm^−1^), nitrogen-rich volatiles (3500–3900 cm^−1^), hydrocarbons (2940 cm^−1^), and CO_2_ (2356 cm^−1^).

Tetrazole plays a synergistic role by generating reactive nitrogen species, favoring crosslinking and C–N/N=N bond formation, producing a denser and more cohesive char. This reinforces the barrier effect of APP. This is corroborated by the increased residual mass and the morphology of the final protective layer.

Thus, the degradation mechanism occurs via synergistic condensed- and gas-phase mechanisms. Tetrazole releases N_2_, diluting gases and promoting crosslinking. APP decomposes into phosphoric acids and volatiles, swelling and reinforcing the char [49]. The combined action produces a protective intumescent barrier, enhancing fire retardancy.

## 4. Conclusions

Five different LDPE/EVA composites containing APP and StVTz were prepared and characterized. Thermal analysis results show that APP and StVTz additives exhibit degradation temperatures that allow their applicability as intumescent additives. The prepared composites exhibit a decrease in Young’s Modulus and an increase in Tensile Strength, as well as elongation at break, attributed to compound formation. In flame retardancy tests, the LDPE/EVA composites containing APP and StVTz exhibited an increase in carbon dioxide residue formation proportional to the additive concentration. Cone calorimeter tests showed that the composites significantly reduced the peak heat release rate and the heat release rate while increasing the residual mass compared to pure LDPE/EVA. These results confirm the effectiveness of the APP/StVTz blend in improving the flame retardancy of the polymer matrix.

In particular, the LDPE/EVA-20-20 composite exhibited the most pronounced flame retardant performance, achieving a 70% reduction in pHRR and forming a dense and compact charred structure. SEM analysis revealed fewer voids and agglomerates in this composite, contributing to a more efficient barrier effect. EDS also indicated a higher oxygen content in the LDPE/EVA-20-20 residue, along with C, N, and P. These results indicate that the formation of a protective layer containing these elements represents the formation of chemical bonds between the elements of the LDPE/EVA resin with APP and StVTz, indicating composition synergism.

The formation of residues containing phosphorus and nitrogen, together with the release of NH_3_ and N_2_ gases, corroborates the proposal that flame-retardant mechanisms act in both the condensed and gas phases.

## Figures and Tables

**Table 1:** Formulation of LDPE/EVA blend and its composites.

Sample	LDPE/EVA	APP	StVTz
LDPE/EVA	100	0	0
LDPE/EVA-10-00	90	10	0
LDPE/EVA-00-10	90	0	10
LDPE/EVA-10-10	80	10	10
LDPE/EVA-15-15	70	15	15
LDPE/EVA-20-20	60	20	20

**Table 2:** TGA results of LDPE/EVA blend and its composites.

Sample	T_d5_ (°C)	T_max_ (°C)	Y_c_ (%)
LDPE/EVA	345	474	6
LDPE/EVA-10-00	327	458	7
LDPE/EVA-00-10	286	440	2
LDPE/EVA-10-10	299	473	11
LDPE/EVA-15-15	297	479	16
LDPE/EVA-20-20	211	465	26

T_d5_: on-set degradation temperature (temperature at 5% weight loss). T_max_: maximum-rate degradation temperature. Y_c_: char yield at 650 °C.

**Table 3:** Thermal properties of LDPE/EVA blend and its composites.

Sample	Cooling			2nd Heating		
	Tc (°C)	ΔHc (J·g^−1^)	X_c_ (%)	Tm (°C)	ΔHm (J g^−1^)	X_c_ (%)
LDPE/EVA	112.7	22.8	7.9	127.5	22.9	7.9
LDPE/EVA-10-00	113.7	17.4	6	127.9	17.1	5.9
LDPE/EVA-00-10	112.2	19.6	6.8	127.5	19.5	6.8
LDPE/EVA-10-10	113.1	13.3	4.6	127.9	13.2	4.6
LDPE/EVA-15-15	113.7	14	4.9	127.1	13.8	4.8
LDPE/EVA-20-20	113.4	10.6	3.7	127.6	10.6	3.7

**Table 4:** Mechanical properties of LDPE/EVA and its composites.

Sample	Young’s Modulus (MPa)	Tensile Strength (Mpa)	Elongation at Break (%)
LDPE/EVA	79.01	14.7	607
LDPE/EVA-10-00	88.37	11.84	561
LDPE/EVA-00-10	99.46	6.62	93
LDPE/EVA-10-10	118.60	7.2	99
LDPE/EVA-15-15	150.35	7.46	65
LDPE/EVA-20-20	213.71	7.7	30

These changes in mechanical properties may influence application suitability.

**Table 5:** Flame retardancy of LDPE/EVA blend and its composites.

Sample	CC Data							UL-94	LOI
	TTI ^a^ (s)	pHRR ^b^ (kW/m^2^)	TpHRR ^c^ (s)	THR ^d^ (MJ/m^2^)	HRR ^e^ (kW/m^2^)	TOF ^f^ (s)	R.M ^g^ (%)		
LDPE/EVA	53	2237	62	68	804	84	0	HB	17
LDPE/EVA-10-00	37	1091	73	65	588	108	8	HB	19
LDPE/EVA-00-10	34	1317	86	75	748	100	0.5	HB	20
LDPE/EVA-10-10	31	853	49	63	601	107	10	HB	20
LDPE/EVA-15-15	32	716	38	68	457	148	16	HB	23
LDPE/EVA-20-20	29	664	61	68	459	150	22	HB	24

^a^ Time to ignition (TTI) is the time needed for the first flame detection. ^b^ Peak heat release rate (pHRR) is the higher value of HRR. ^c^ Time to pHRR (TpHRR) is the time required to achieve the pHRR. ^d^ Total heat release (THR) is the total heat released by the sample during the entire duration of the test. ^e^ Average heat release rate (HRR) is the irradiated thermic power released by the sample per square meter. ^f^ Time of flame (TOF) is the flame lifetime. ^g^ Residual mass (R. M.) is the amount of residue in percentage.

**Table 6:** EDS of LDPE/EVA blend and its composites.

	Wt. (%)		
Element	LDPE/EVA-10-10	LDPE/EVA-15-15	LDPE/EVA-20-20
C	10.26	20.71	7.35
N	1.17	1.83	1.38
O	21.89	19.25	33.45
P	66.69	58.21	57.82

## Data Availability

All information about the work is available in this manuscript and in the supplementary file. Further inquiries, please contact the corresponding author directly.

## Supplementary Materials

The following supporting information can be downloaded at https://www.mdpi.com/article/10.3390/polym17212933/s1. Figure S1: Infrared spectrum of EstAcn precursor copolymer and SVT copolymer; Figure S2: 1H-NMR (a), 13C-NMR (b), spectra of EstAcn and SVT; Figure S3: TGA (a) and DTG (b) curves under air of EstAcn and SVT copolymers; Figure S4: DSC curves of of EstAcn and SVT.
