# Supplementary material for: Flame-Retardant Properties of a Styrene-Vinyl Tetrazole Copolymer Additive in an LDPE/EVA Blend
Source: Polymers (Basel). 2025 Oct 31;17(21):2933. doi: 10.3390/polym17212933 (PMC12608187; doi:10.3390/polym17212933)
Supplement: Supplementary file 1 [file polymers-17-02933-s001.zip › polymers-3922132-supplementary.pdf]

Flame-retardant properties of a styrene-vinyl tetrazole copolymer additive in an LDPE/EVA blend

Karla Fabiola Rodríguez Ramírez[a], Jesús Francisco Lara Sánchez[a], Orlando Castro Reyna[a], Pedro Espinoza Martínez[a], Jesús Alejandro Espinosa Muñoz[a], José David Zuluaga Parra[b], Rachel Faverzani Magnago[a], Saul Sanchez Valdés[a], Luciano da Silva[a,*]

[a] Applied Chemistry Research Center, Department of Transformation Processes, Blvd. Enrique Reyna H. 140, 25294 Saltillo, Coah, Mexico

[b] Rothamsted Research, West Common, Harpenden, Hertfordshire, United Kingdom

[c] Programa de Pós-Graduacao em Biotecnologia e Biociencia, Universidade Federal de Santa Catarina, Trindade, 88040-900, Florianópolis, Brasil

SUPLEMENTARY DATES

INDEX OF FIGURES

Figure S1. Infrared spectrum of EstAcn precursor copolymer and SVT copolymer…………………………………………………………….………….. 3

Figure S2. ^{1}H-NMR (a), 13C-NMR (b), spectra of EstAcn and SVT…………… 4

Figure S3. TGA (a) and DTG (b) curves under air of EstAcn and SVT copolymers…………………………………………………………………... 5

Figure S4. DSC curves of of EstAcn and SVT………………………………….. 6

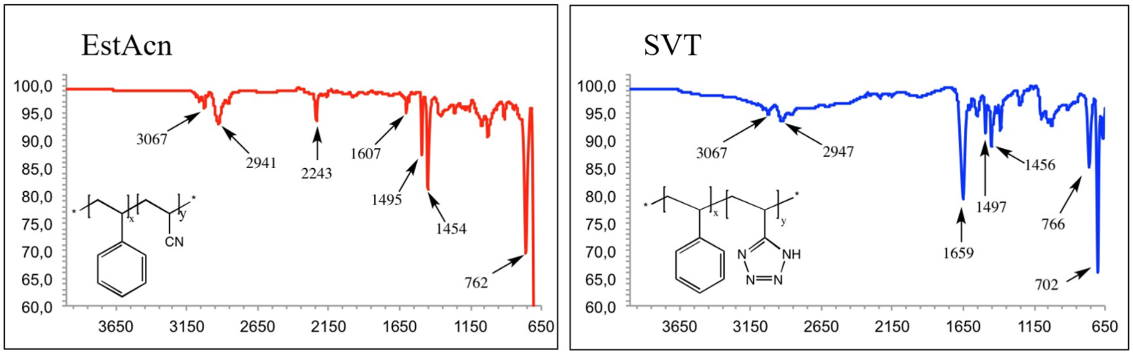

Figure S1. Infrared spectrum of EstAcn precursor copolymer and SVT copolymer.

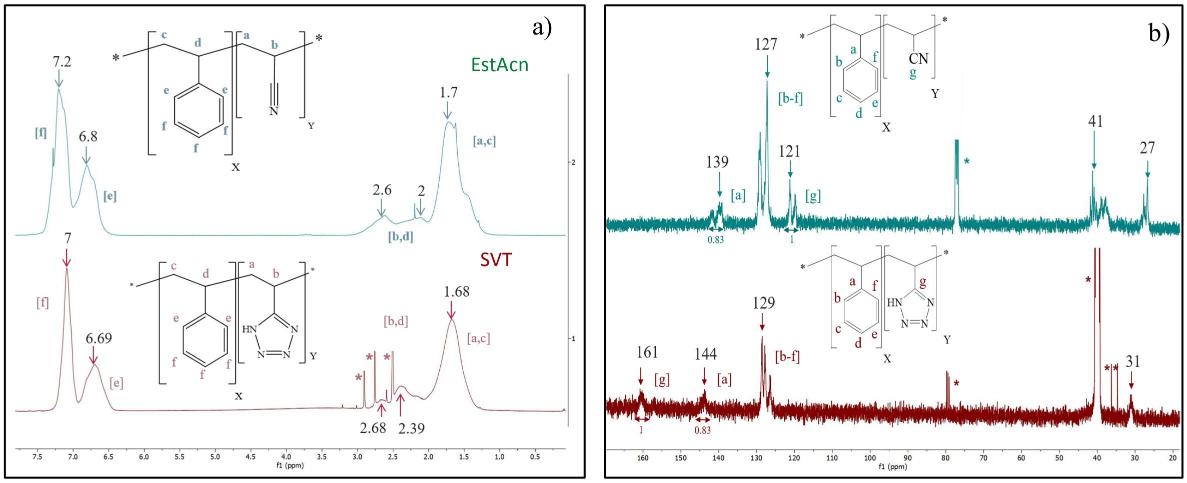

Figure S2. ^{1}H-NMR (a), 13C-NMR (b), spectra of EstAcn and SVT.

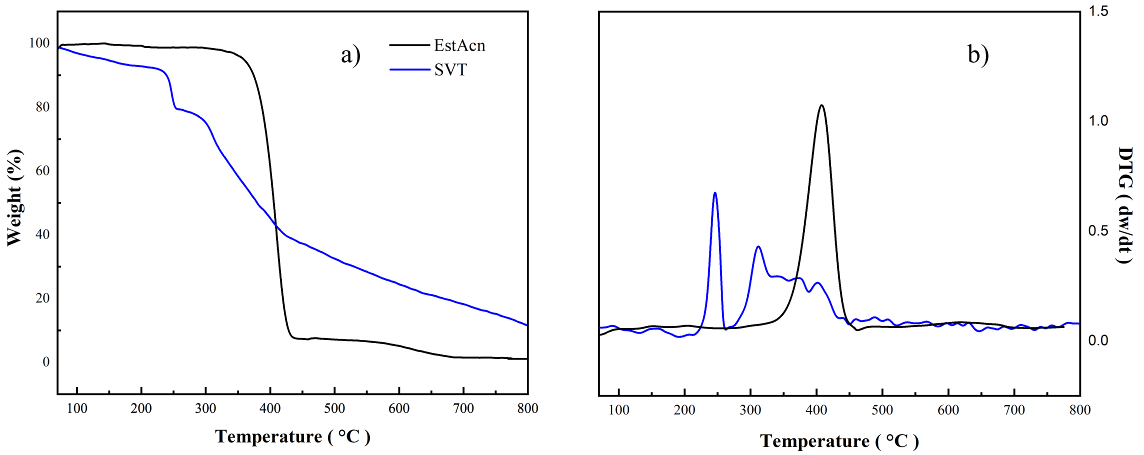

Figure S3. TGA (a) and DTG (b) curves under air of EstAcn and SVT copolymers.

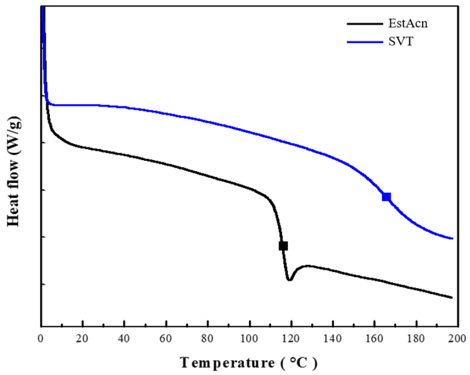

Figure S4. DSC curves of of EstAcn and SVT.
